# *Acremonium (Sarocladium)* periprosthetic joint infection: case report, literature review, and proposed antifungal regimen

**DOI:** 10.1186/s12879-022-07824-2

**Published:** 2022-12-29

**Authors:** Nikko Rowe A. Tabliago, David Maish, Raquel M. Martinez, Mark A. Shelly, Bridget Yablonski, Taesung Kwon

**Affiliations:** 1 Department of Internal Medicine, Geisinger Medical Center, 100 North Academy Avenue, Danville, PA 17822 USA; 2 Department of Orthopedics, Geisinger Medical Center, Danville, PA USA; 3 Department of Laboratory Medicine, Geisinger Medical Center, Danville, PA USA; 4 Department of Infectious Diseases, Geisinger Medical Center, Danville, PA USA; 5 Department of Infection Prevention and Control, Geisinger Medical Center, Danville, PA USA

**Keywords:** Acremonium, Periprosthetic joint infection, Antimicrobial therapy, Voriconazole, Case report

## Abstract

**Background:**

Fungal periprosthetic joint infections are rare. *Acremonium* osteoarticular infections are scarcely reported. Variable susceptibility to antifungal agents have been reported and optimal pharmacotherapy has yet to be established. Here we illustrate an *Acremonium* osteoarticular infection involving a prosthetic joint and present an antifungal regimen that had led to treatment success.

**Case presentation:**

A 60-year-old female with a body mass index (BMI) of 40 had left total knee arthroplasty done in 2012 with a cementless implant for knee osteoarthritis. In 2019, the patient had asymptomatic, progressive osteolysis with fracture and migration of the femoral component warranting replacement. Eleven months later, the patient developed significant pain, redness, and swelling in the left leg and knee concerning for periprosthetic joint infection that failed outpatient antibiotic treatment. Further investigation revealed infection by *Acremonium* species. A revision of the joint was successfully completed, and the patient was placed on voriconazole for one year. Subsequent cultures did not yield any fungal growth.

**Conclusion:**

While an optimal antifungal regimen for periprosthetic joint infections has not been well established, voriconazole is a relatively safe and effective agent that can be used as a long-term therapy. With variable susceptibility testing in reported isolates, individualized antifungal susceptibility should be used to guide therapy for *Acremonium* infections.

## Background

Fungal infections of prosthetic hip and knee joints are uncommon, comprising about 1% of prosthetic joint infections [1]. Diagnosis is difficult because of the lack of accepted guidelines. Delayed diagnosis and inappropriate treatment can result in significant morbidity for these patients. As such, *Acremonium* has been garnering notoriety for focal infections in otherwise healthy individuals as well as systemic disease.

*Acremonium* infections have been implicated in many organ systems, including the eyes, lungs, and the gastrointestinal tract with mycetoma being the most common type of *Acremonium* infection. Traumatic inoculation has been described as the likely cause of this fungal infection, and the local infection in the immunocompetent is indeed more prevalent than systemic infection in the immunocompromised [2]. Throughout this report, *Acremonium* will be referenced; however, it is important to note there has been a shift in nomenclature of *Acremonium* towards *Sarocladium* as modern phylogenetics is implemented. Many of *Acremonium* species have been found to be of the genus *Sarocladium*, which necessitates further studies to be done on isolates currently defined as species of *Acremonium* [3].

Only a handful of cases describe a bone or joint infection. One of the earliest cases involved a child that developed septic arthritis, which resulted in delayed recognition of the culprit pathogen, as it was deemed to be a contaminant [4]. Here we illustrate an *Acremonium* osteoarticular infection involving a prosthetic joint and present a potential antifungal regimen.

## Case presentation

A 60-year-old female with a body mass index (BMI) of 40 had left total knee arthroplasty done in 2012 with a cementless implant for knee osteoarthritis. Asymptomatic, progressive osteolysis was seen in the distal femur in 2016 and subsequently resulted in medial condyle fracture with the superior migration of the femoral component by February 2019. In December 2019, the patient had a distal femur replacement which was functional but minimally painful. Eleven months later, the patient underwent an unrelated laryngeal surgery and developed significant pain, redness, and swelling in the left leg and knee shortly after the procedure. Pain, rated 7 out of 10 on pain scale, worsened with flexion and extension of the knee resulting in an antalgic gait and use of a cane.

She presented to the office 19 days after her surgery. On examination, her blood pressure was 132/80 mmHg, pulse 92 beats per minute, temperature 36 °C, respirations 20 breaths per minute. There was a punctate opening superolateral to the left patella with purulent drainage, and it was sent for aerobic and anerobic bacterial culture analysis but did not yield any growth. No additional labs were performed at this time. A course of trimethoprim-sulfamethoxazole was prescribed for empiric treatment of presumed cellulitis, and significant improvement and resolution of drainage were observed; however, the pain did not resolve and the duration was extended to three weeks. Observation after completion of antibiotic therapy revealed gradually worsening edema.

The peripheral blood white blood cell (WBC) count was 8780 cells/µL, hemoglobin 9.0 g/dL, platelets 519,000 cells/µL, erythrocyte sedimentation rate (ESR) 27 mm/h, and C-reactive protein (CRP) 6 mg/L. The arthrocentesis revealed a synovial WBC count of 5440 cells/mm^3^: predominantly neutrophils (89%). Synovial fluid CRP was 0.5 mg/L with alpha defensin detected. Additionally, the synovial fluid fungal culture grew *Acremonium* species. Synovial gram stain and bacterial culture were negative.

**Fig. 1:**
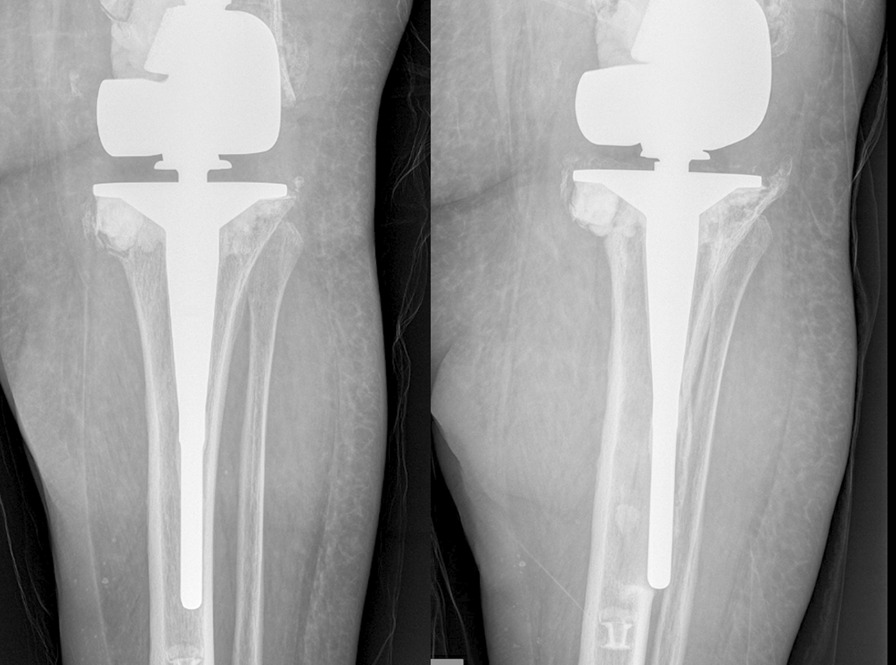
X-rays left knee. One year progression of failed left knee tibial component with chronic penetration of the lateral tibial cortex evident by neocortex and reactive changes

The tibial component had failed in December 2020 (Fig. 1) with the long-stemmed tibial component protruding through the lateral cortex and reactive bone formation. Erosion was noted around the implant distally with intact proximal positioning. The left knee hardware was removed with placement of static antibiotic spacer containing vancomycin and tobramycin.

Four of the six intraoperative cultures again grew *Acremonium* in the hardware sonicate and bone samples.

Table 1 demonstrates the results of the antifungal susceptibility testing performed by a reference laboratory. The patient was started on voriconazole 400 mg orally twice daily for two doses, followed by maintenance voriconazole 200 mg twice daily and then discharged. Upon outpatient follow-up, the patient was tolerating therapy without adverse reactions, voriconazole levels were < 0.5 µg/mL, and the dose was increased to 300 mg orally twice daily. The follow-up levels were 1.0–1.5 µg/mL. Approximately 3.5 months after explantation, the distal femur replacement was reimplanted per patient’s preference and intraoperative cultures of the synovial fluid, tissues, and hardware sonicate culture did not grow any bacteria or fungus. Overall, the patient completed one year total of antifungal therapy.

**Table 1:** *Acremonium* susceptibilities from synovial fluid culture from surgery

Antifungal	MIC (µg/mL)
Amphotericin B	1.0
Fluconazole	32.0
Itraconazole	1.0
Voriconazole	1.0
Isavuconazole	8.0

## Discussion and conclusions

Fungal periprosthetic joint infections (PJIs) have been reported to comprise only 1–3% of all joint infections, the majority of which are attributed to *Candida* and *Aspergillus* species [1]. Also, it is widely accepted that fungal infections are a hallmark illness of immunocompromised patients. However, it has been reported that nearly 40% of fungal osteoarticular infections are in apparently immunocompetent patients [5]. As with any unusual pathogen, cases should be reviewed for commonalities from services provided during the care of the patient to ensure no potential for epidemiological linkage within the healthcare system. No other causative factor was delineated in our case other than her traumatic fall ten years prior to her presentation. The occult infection dwelled over many years and destroyed the bone to the point where it could not support the prosthesis and subsequently led to component failure. Interim replacement without identifying the infection resulted in repeat failure of the new prosthesis in diseased bone. Reviewing prior orthopedic cases at our institution that involved culture findings with *Acremonium*, most have been ruled as a contaminant based on the scarcity of fungal growth (i.e., fungal growth in only one out of several cultures) and lack of other symptoms, signs, and laboratory evidence of active infection.

The diagnosis of a PJI relies on a combination of clinical presentation, laboratory data, and intraoperative findings [6]. This includes visualization of a communicating sinus tract or purulence, a pathogen isolated by culture from at least two separate tissue or fluid samples, and histopathologic inflammation of periprosthetic tissue. Efforts have been made to introduce preoperative diagnostic criteria improving one’s pretest probability with the inclusion of serum inflammatory markers and synovial fluid analysis [7]. These weighted minor criteria propose a likelihood of infection. *Acremonium* isolates can be difficult to identify when solely using morphological methods. To further define the isolate, molecular confirmation via sequencing of the internal transcribed spacer (ITS) fungal barcode and the actin gene can be performed. However, speciation is not routinely done in the clinical microbiology laboratory. Two species, *Acremonium kiliense* (now *Sarocladium kiliense*) and *Acremonium egyptiacum*, have been proven to be involved in human infections [8]. Although it was not thought to be clinically important at the time, identification of the species would have been an opportunity to align our isolate with one of these known pathogens.

*Acremonium* is an uncommon human pathogen. *Acremonium* osteoarticular infections are reported even less so in the literature. In a review of 61 fungal osteoarticular infections, *Acremonium* was noted in 3 cases, none of which involved prosthetic joints [9]. A combination of surgery and antifungals is imperative to provide the patient with best chance of success. After surgical revision, an extended course of antifungals is recommended. For *Candida* and *Aspergillus* PJIs, this can range from 3 to 6 months depending on the host response. Wu et al. described, to the best of our knowledge, the first PJI involving *Acremonium* along with *Penicillium*. The patient in the study had their hardware removed and was placed on fluconazole for 12 months [10]. It was shown previously that the most effective agents for *Acremonium* based on the minimum inhibitory concentration (MIC) and the minimum fungicidal concentration (MFC) were amphotericin B, itraconazole, and ketoconazole [11]. More recently, Perdomo et al. reported that terbinafine, posaconazole, and amphotericin B were the most effective, but variable susceptibility makes it difficult to choose the optimal antifungal agent [2]. Since there are no standardized MIC breakpoints for *Acremonium*, it was particularly important to choose an agent with commercially available monitoring methods while susceptibility testing was pending. For our patient, we empirically started voriconazole based on reported cases of its use for *Acremonium* infections in different body sites and known bioavailability in bone and synovial fluid [12]. Also, serum voriconazole concentrations can be monitored for dose adjustments. A relatively low MIC to voriconazole has been reported (0.25 µg/mL), at which 90% of *Acremonium* isolates were inhibited [13]. In previous studies, achieving a voriconazole trough concentration of > 1 µg/mL has shown improved outcomes for susceptible organisms but a concentration of < 0.25 µg/mL has a higher probability of treatment failure [14]. Toxicity—manifested by visual disturbances (photopsia), liver dysfunction, skin reaction, and neurotoxicity (confusion and visual hallucinations)—is usually not noted until the concentration reaches levels of 4–6 µg/mL [14]. Optimal trough levels for efficacy have yet to be established. Our *Acremonium* MIC to voriconazole was 1.0 µg/mL. Our patient’s initial trough concentrations were < 0.5 µg/mL but brought to 1.0–1.5 µg/mL with dose adjustment, leading to treatment success.

*Acremonium* is becoming recognized as an emerging pathogen. Fungal PJI is rare, more so with *Acremonium*, and a high index of suspicion and familiarity with diagnostic criteria are key to recognizing it. While it may often be dismissed, our case shows that *Acremonium* grew in multiple cultures: synovial fluid, hardware sonicate, and bone. This puts forward robust evidence for a genuine fungal infection. The choice of an antifungal regimen has not been well established for prosthetic joint infection. We have presented a relatively safe and effective antifungal agent which can be used as a long-term therapy. As there is only one other report of *Acremonium* PJI, there is limited data with which to compare. With variable susceptibility testing in reported isolates, individualized antifungal susceptibility should be used to guide therapy for *Acremonium* infections [2, 11]. The source of this infection for our patient remains unclear.

## Data Availability

Not applicable.

## Abbreviations

BMI: Body mass index
CRP: C-reactive protein
ESR: Erythrocyte sedimentation rate
ITS: Internal transcribed spacer
MFC: Minimum fungicidal concentration
MIC: Minimum inhibitory concentration
PJI: Periprosthetic joint infection
WBC: White blood cell
